# Predicting RF Heating of Conductive Leads During Magnetic Resonance Imaging at 1.5 T: A Machine Learning Approach

**DOI:** 10.1109/EMBC46164.2021.9630718

**Published:** 2021-11

**Authors:** Can Zheng, Xinlu Chen, Bach T. Nguyen, Pia Sanpitak, Jasmine Vu, Ulas Bagci, Laleh Golestanirad

**Affiliations:** Department of Electrical Engineering, Northwestern University, Evanston, IL 60208 USA.; Department of Electrical Engineering, Northwestern University, Evanston, IL 60208 USA.; Department of Radiology, Northwestern University Chicago, IL 60611 USA.; Department of Biomedical Engineering, Northwestern University, Evanston, IL, 60608 USA.; Department of Radiology, Northwestern University Chicago, IL 60611 USA.; Department of Radiology, Northwestern University Chicago, IL 60611 USA.; Department of Radiology and Department of Biomedical Engineering, Northwestern University, Chicago, IL, 60611 USA

## Abstract

The number of patients with active implantable medical devices continues to rise in the United States and around the world. It is estimated that 50-75% of patients with conductive implants will need magnetic resonance imaging (MRI) in their lifetime. A major risk of performing MRI in patients with elongated conductive implants is the radiofrequency (RF) heating of the tissue surrounding the implant’s tip due to the antenna effect. Currently, applying full-wave electromagnetic simulation is the standard way to predict the interaction of MRI RF fields with the human body in the presence of conductive implants; however, these simulations are notoriously extensive in terms of memory requirement and computational time. Here we present a proof-of-concept simulation study to demonstrate the feasibility of applying machine learning to predict MRI-induced power deposition in the tissue surrounding conductive wires. We generated 600 clinically relevant trajectories of leads as observed in patients with cardiac conductive implants and trained a feedforward neural network to predict the 1g-averaged SAR at the lead tips knowing only the background field of MRI RF coil and coordinates of points along the lead trajectory. Training of the network was completed in 11.54 seconds and predictions were made within a second with R^2^ = 0.87 and Root Mean Squared Error (RMSE) = 14.5 W/kg. Our results suggest that machine learning could provide a promising approach for safety assessment of MRI in patients with conductive leads.

## I. Introduction

Magnetic resonance imaging (MRI) expanding rapidly in the indications for a wide variety of neurological, cardiac, and musculoskeletal diseases thanks to its non-invasive properties and unrivaled soft tissue contrast. However, MRI is not easily accessible to a sizeable cohort of patients with conductive medical implants, like the cardiovascular implantable electronic devices (CIEDs) or deep brain stimulation (DBS) systems. The major safety hazard of MRI in patients with CIEDs or DBS systems is due to the antenna effect, where the electric field of MRI scanner couples with implanted leads and amplifies the specific absorption rate (SAR) of radiofrequency energy in the tissue surrounding the tip of implanted lead [1-4]. Full-wave electromagnetic simulations are routinely applied to characterize the interaction of MRI RF fields with the human body in the presence of conductive implants to quantify RF heating of the implant while accounting for effects of MRI coil’s geometry [5-11], implant’s structure, material and trajectory [12, 13], as well as patient’s body composition [14]. These simulations, however, are notoriously cumbersome in terms of computational time and memory requirements: even taking advantage of today’s high-power computing clusters, it typically takes tens of hours to complete a single simulation scenario with enough degrees of complexity to provide good agreement with physical measurements [15].

Novel machine learning methods have been recently proposed as a paradigm shift in the assessment of MRI RF heating. Pioneering work by Chen group has shown that neural networks can predict the worst-case heating of orthopedic fixation plates in MRI environment when only the knowledge of implant’s geometrical features is at hand [16, 17]. In their work, however, the implant’s position was predetermined within the MRI RF coil and thus, the effect of variation in electric field exposure due to changes in implant’s location and orientation was not investigated. This is particularly important in the assessment of RF heating of elongated conductive implants, such as leads in CIEDs, as lead’s trajectory within the human body and its orientation with respect to MRI RF fields substantially affect RF heating [10, 11, 18-20].

In this work, we investigated whether neural networks could predict the 1g-averaged SAR at tips of implanted leads in a human body phantom exposed to MRI RF radiation at 63.6 MHz (proton imaging at 1.5 T). We created lead models with clinically relevant trajectories and positions corresponding to what is observed in patients with cardiac pacemakers. We then trained a feedforward neural network to predict the maximum of 1g-averaged SAR in the tissue surrounding the tip of the lead directly from coordinates of points along the lead trajectory. The performance of the network is discussed with regard to different trajectories.

## II. METHODS

### A. Design of Lead Trajectories

**Figure 1.**
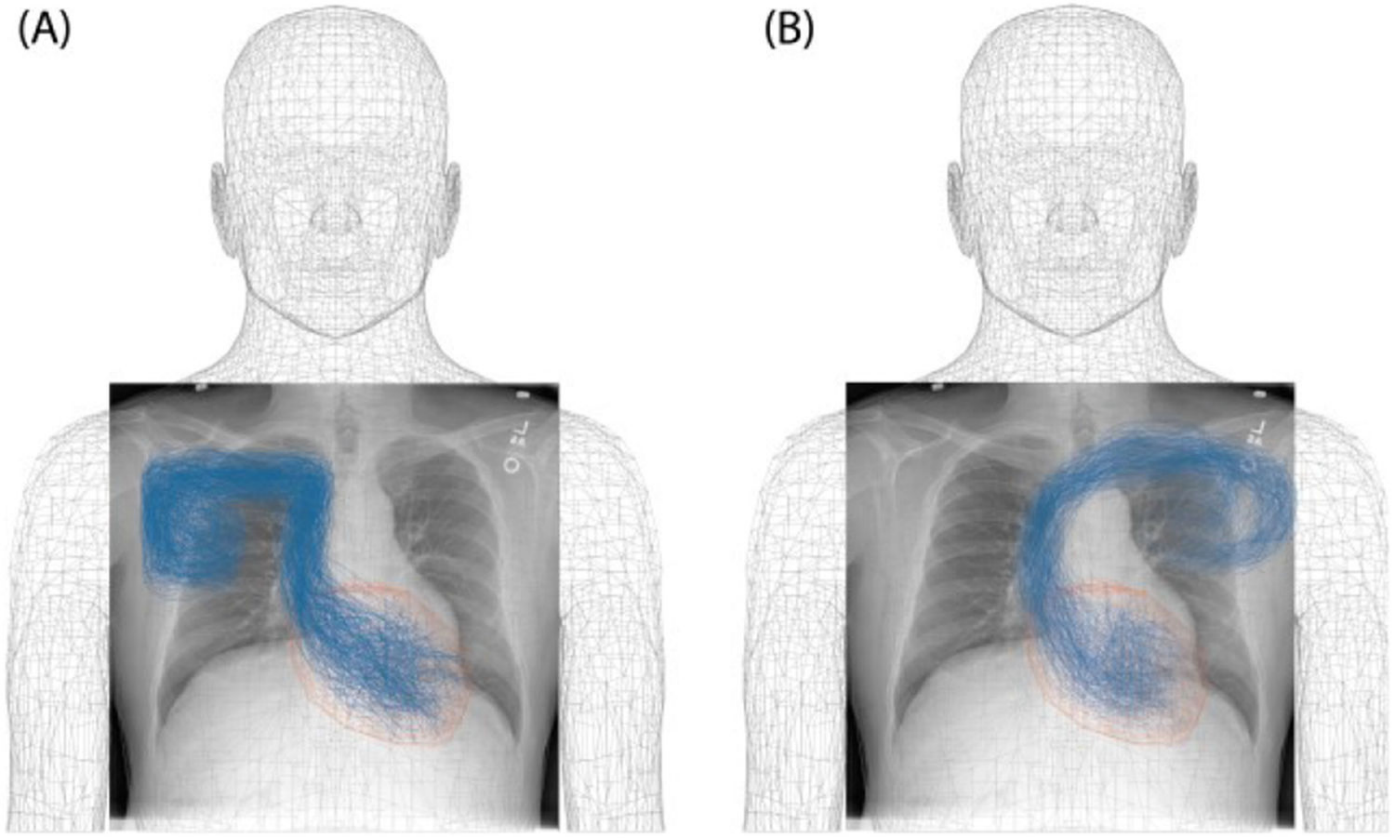
Example of X-ray photograph of a patient with CIED overlaid on ANSYS human body model and manual trajectories with IPGs on right (A) as well as left (B).

We generated 600 clinically relevant lead trajectories based on the evaluation of thoracic X-ray photographs of patients with cardiac pacemakers and defibrillators. From these, 300 trajectories corresponded to cases where the implanted pulse generator (IPG) was in the left pectoral region and 300 trajectories corresponded to the IPG in the right pectoral region. All trajectories were 58 cm long, similar to typical active fixation leads (Medtronic 5076, Medtronic 4076) and passive fixation leads (Medtronic 4074) for cardiac pacing [21]. Lead trajectories were generated in Rhino3D^®^ (Robert McNeal and Associates, Seattle, WA) using the Grasshopper module. ANSYS human body model (ANSYS Human Body Model V3, accessed 2020) [22] was used for anatomical guidance. We designed trajectories starting from the assumptive position of IPG in left or right pectoral region and followed a path through the subclavian vein or superior vena cava to the heart. To generalize the model for different body forms, the size of the heart was enlarged by 50%. From our inspection of patients’ radiographic images, as well as reports from other groups [18], we found that the lead trajectories in large veins had subtle differences whereas the distal part in the pectoral regions and the position of the lead tips had significant variation, virtually covering the entire heart. These guidelines were incorporated in the algorithm that generated lead trajectories as can be observed in Fig. 1.

### B. Numerical Modeling

**Figure 2.**
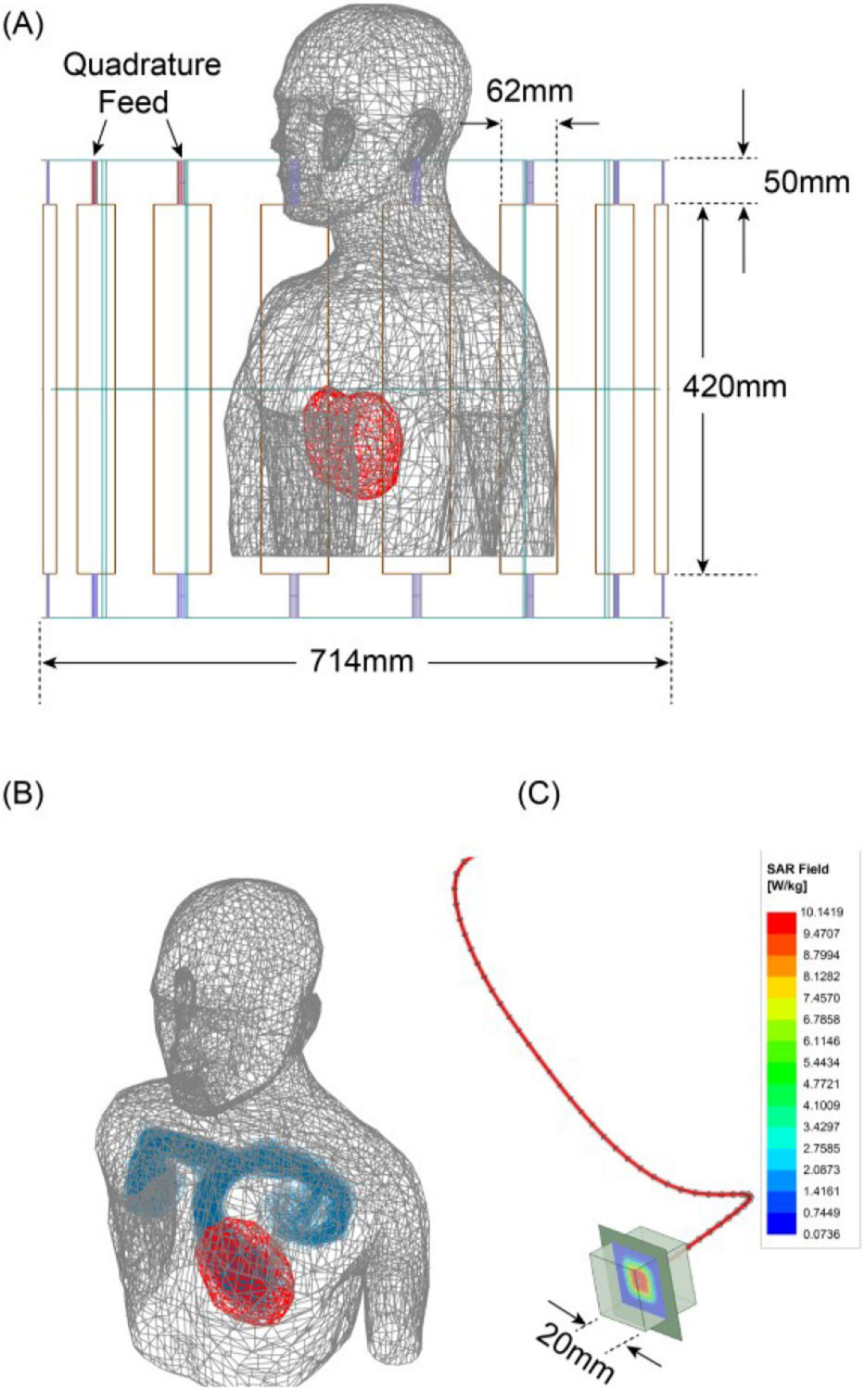
(A) Simulation setup in ANSYS HFSS showing homogeneous body model and MRI RF coil. The heart is shown to visualize the position of distal parts of leads and was not included in FEM simulations (B) Overlay of 600 trajectories in the body model (C) 1g-averaged SAR on a central axial plane within the 20 × 20× 20 *mm*^3^ cube surrounding the exposed lead’s tip.

Finite element simulations (Fig. 2) were performed to calculate the local SAR at tips of implanted lead models during MRI RF exposure using ANSYS Electronics Desktop 2019 R2 (ANSYS, Canonsburg, Pennsylvania, USA). A model of a low-pass 16-rung birdcage coil (diameter = 714 mm, length = 470 mm) was created and tuned to its resonance frequency at 63.6 MHz corresponding to proton imaging at 1.5 T. The coil was loaded with a homogeneous human body model (ANSYS Human Body Model V3, accessed 2020) with electric properties of average tissue (σ = 0.47 *s/m*, ϵ_*r*_ = 80). Lead trajectories were imported from Rhino3D and modeled as 90%:10% platinum-iridium wires (Pt:Ir, σ = 4 × 10^6^ *s/m*, diameter = 1 mm) wrapped within urethane insulation (ϵ = 3.5, diameter = 2 mm) with 2 mm exposed tip. To increase the accuracy of numerical simulations around the lead’s tip, we defined a 20 × 20 × 20 *mm*^3^ cubic region in which we assigned a fine mesh resolution (RMS element length = 1.57 mm). The input power of the coil was adjusted such that the spatial mean of $B_{1}^{+}$ on a transverse plane passing through the center of the coil was 2 μT. The maximum of 1g-averaged SAR (*MaxSAR*_1*g*_) within the high-resolution mesh region surrounding the tip was calculated with the HFSS built-in SAR module and used as the ground truth to train the neural network.

### C. Feedforward Neural Network Architecture

The total data for 600 trajectories was divided into training set (64%), validation set (16%) and testing set (20%). A feedforward neural network was designed, which contained one input layer, five hidden layers with dropouts and one output layer. The x, y, z coordinates of 116 points sampled along the length of each lead were first concatenated to 348 × 1 from 116 × 3 as the input of the neural network. The output would be the prediction of *MaxSAR*_1*g*_ in the cubic region surrounding the lead’s tip. Fully connected hidden layers activated by ReLU function were used to learn the nonlinear relationship between lead coordinates and *MaxSAR*_1*g*_. To reduce overfitting and improve generalization error [23], a dropout was introduced for each hidden layer. Finally, the output layer linearly regressed the predicted *MaxSAR*_1*g*_ as one scaler.

**Figure 3.**
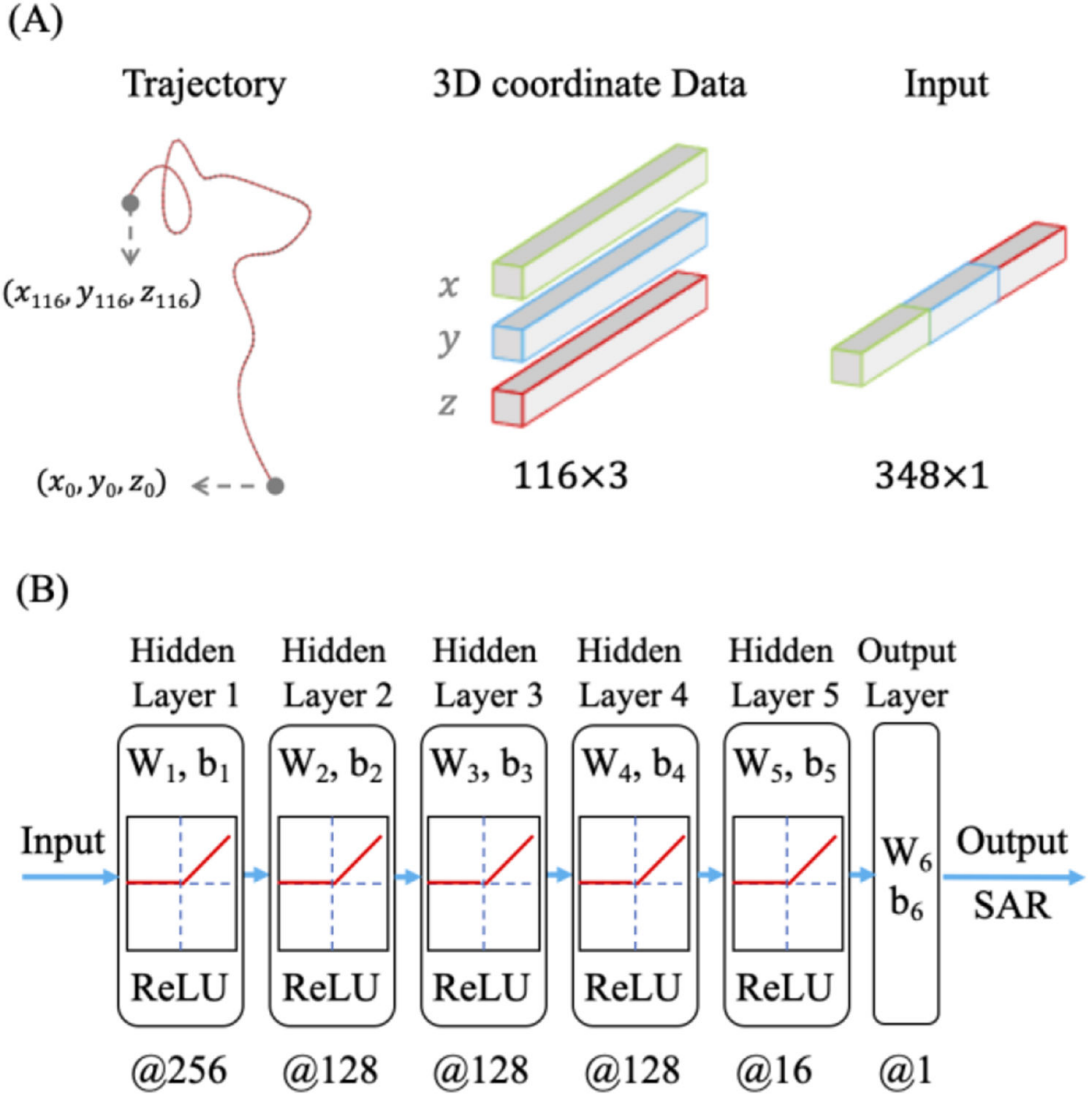
(A) Concatenation of 3D coordinate. (B) Structure of feedforward neural network; *W*_*i*_ and *b*_*i*_ represent weight and bias matrices for each layer; @ is followed by the number of neurons of every layer.

Hyperparameters including the number of neurons, learning rate, and dropout rate were tuned by Ray Tune— a Python library that accelerates hyperparameter tuning with parallelized computing. The search algorithm was Bayesian Optimization and Hyperband (BOHB), an algorithm that combines Hyperband with Bayesian optimization and is dominant in both efficiency and performance [24]. As a result, the number of neurons of five hidden layers were optimized to 256, 128, 128, 128 and 16 respectively (Fig. 3).

## III. RESULTS

### A. Simulation Results: Convergence

ANSYS HFSS followed an adaptive mesh scheme where an initial mesh with a user-defined resolution (20 mm in human body, 2 mm in cubic region, 2 mm in lead’s insulation, 0.5 mm on lead’s wire, and 10 mm on the coil) was seeded. Mesh resolution was enhanced at each adaptive pass until the maximum difference between iterative scattering parameters fell below a predefined threshold of 0.02. All 600 simulations converged within 3 adaptive passes. Table 1 gives the mesh statistics for a representative simulation. Each simulation took around 90 minutes to complete on a DELL PowerEdge R740 server with 1.5 TB memory and 2xXenon(R) Gold 6140 CPUs each having 18 processing cores.

### B. Simulation Results: SAR Distribution

**Figure 4.**
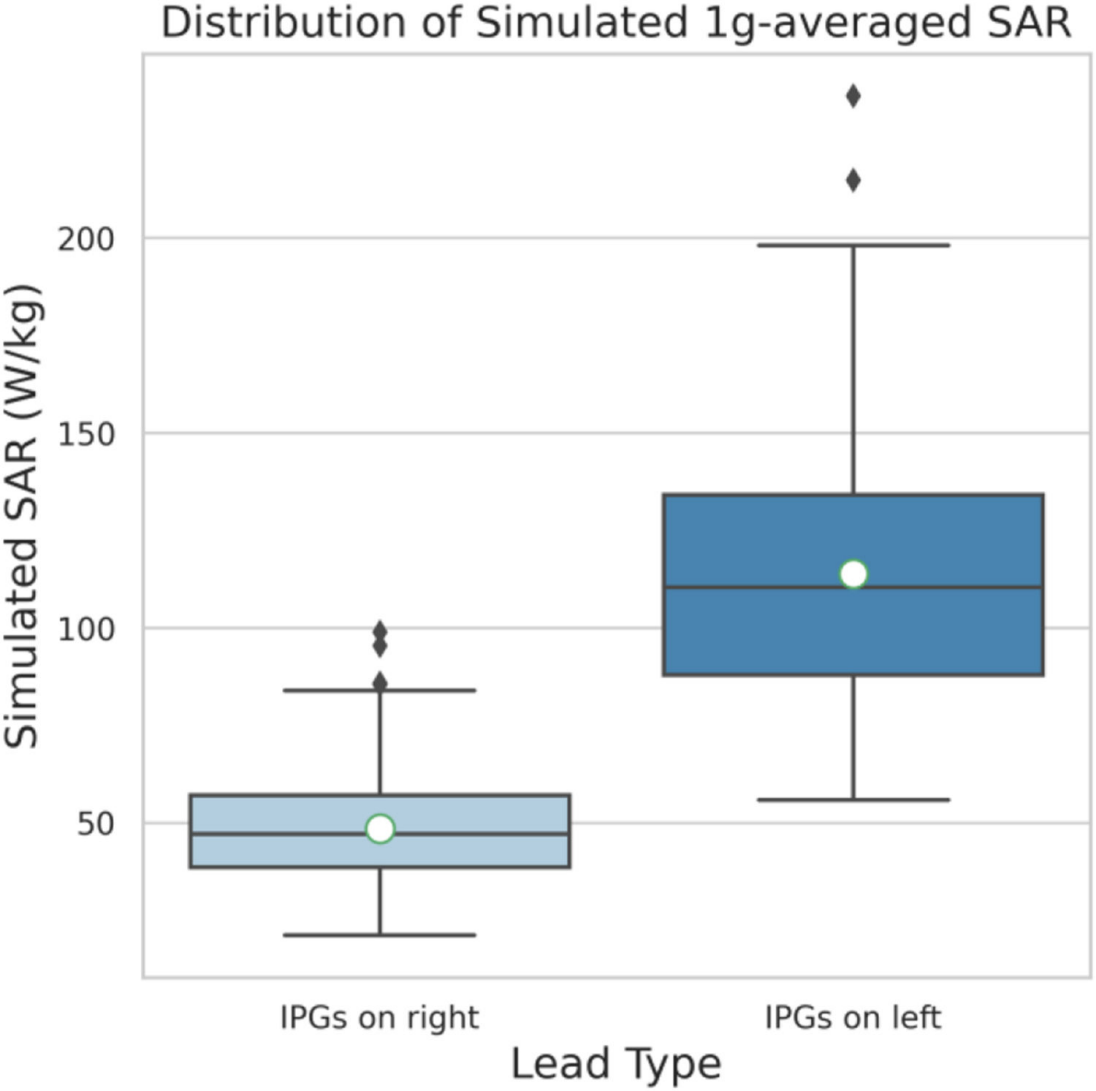
Distribution of Simulated 1g-averaged SAR with IPGs in right as well as in left pectoral regions. Circles indicate the mean values.

Fig. 4 gives the distribution of the normalized *MaxSAR*_1*g*_ values for trajectories with IPGs in right and left pectoral regions. *MaxSAR*_1*g*_ was 48.67 ± 14.49 *W/kg* and 113.97 ± 32.28 *W/kg* for trajectories with IPG in right and left pectoral regions respectively. A one-tail t-test showed the *MaxSAR*_1*g*_ of trajectories with IPG in left side to be significantly greater ( *p* = 3 × 10^−114^ ) than SAR of trajectories with IPG in the right pectoral region.

### C. Neural Networks based SAR predictions

**Figure 5.**
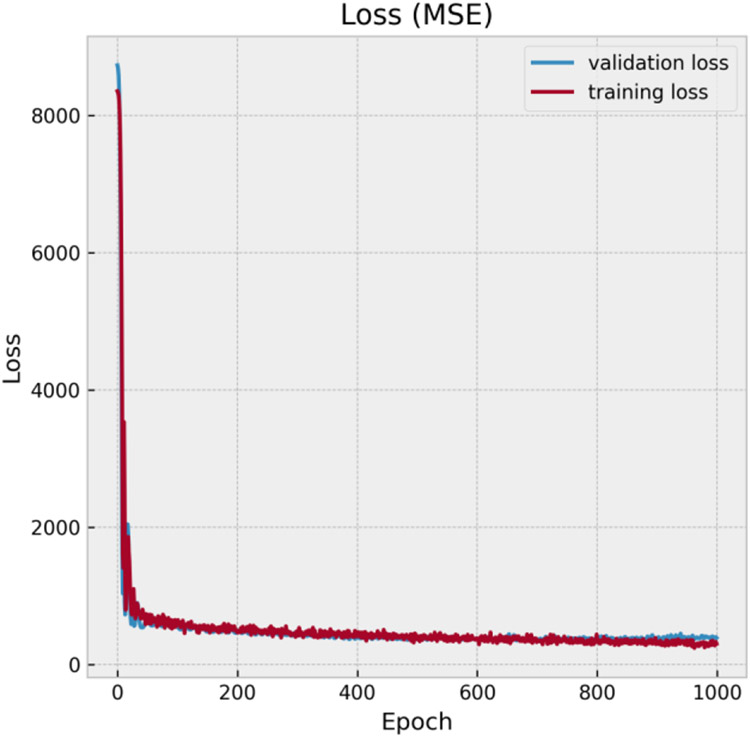
Training loss and validation loss with increasing epochs

Mean squared error (MSE) was chosen to be the optimization target during training. Both training and validation losses substantially decreased within 100 epochs and converged to ~ 380 *W*^2^*/kg*^2^ after 700 epochs (Fig. 5). After that, the network started to overfit the training data as the gap between validation loss and training loss increased. Therefore, the number of epochs for training was set to 700. On the test dataset, the Root Mean Squared Error (RMSE) is 14.5 *W/kg*, for trajectories with IPGs in right and left pectoral regions were 9.2 *W/kg* and 18.3 *W/kg* respectively.

**Figure 6.**
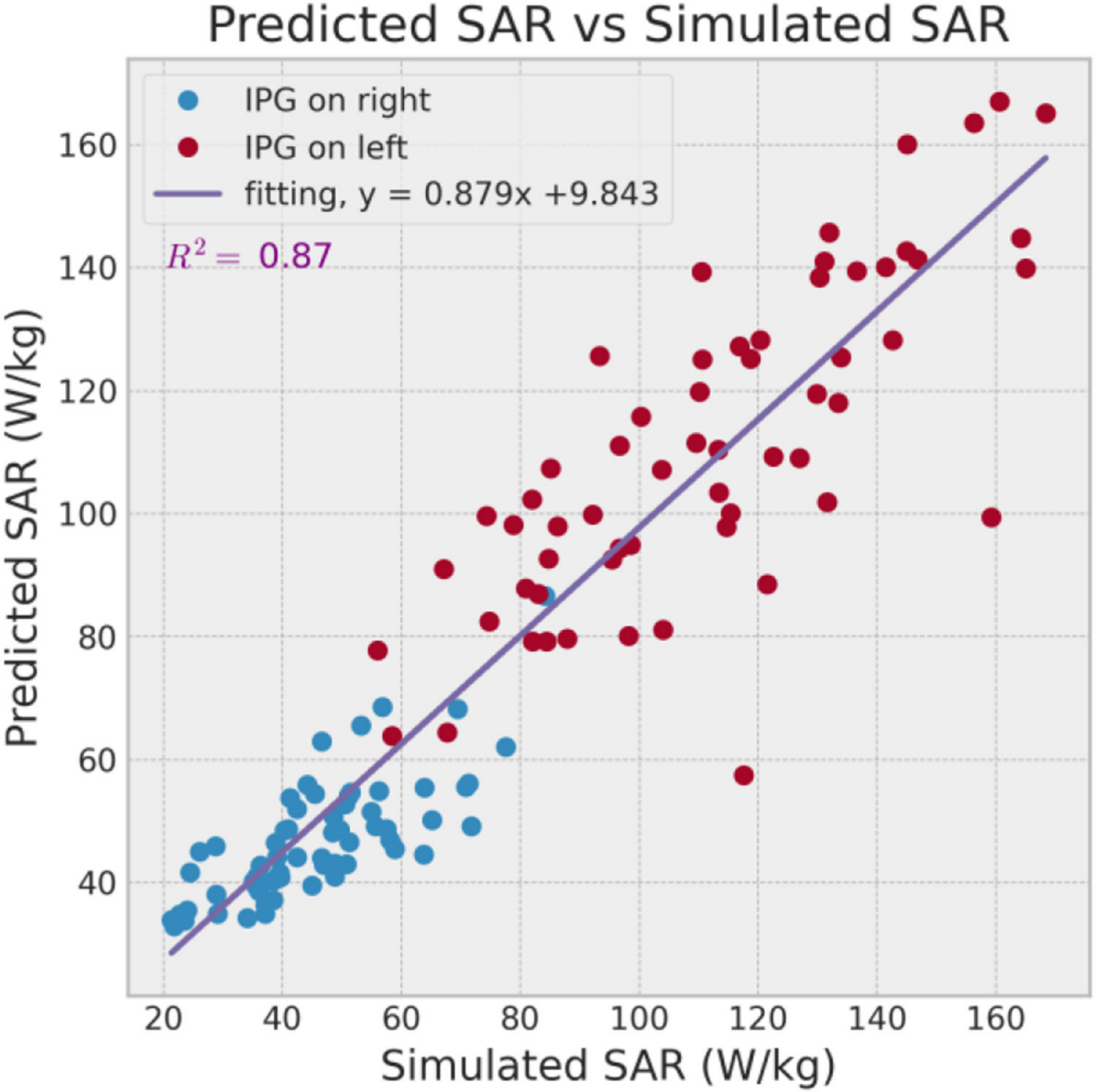
Performance of the feedforward neural network with predicted 1g-averaged SAR vs simulated 1g-averaged SAR. The coefficient of determination (R^2^) was relatively high (equals to 0.87).

Fig. 6 shows the comparison of simulated and predicted *MaxSAR*_1*g*_ resulting a relatively high R^2^ score of 0.87. The feedforward network performed better in predicting the heating of trajectories with IPGs in right than that in left, but the latter still maintained enough linearity in a wider SAR range with few outlier predictions.

## IV. Discussion and Conclusion

MRI is refuted to a sizeable cohort of patients with conductive implants because of its safety hazards according to the RF-induced heating of tissue surrounding the implant. Pre-assessment of heating is essential to determine the risk/benefit ratio of MRI exams in these patients and is typically performed through phantom experiments or full-wave electromagnetic simulations both of which being substantially time-consuming. Machine learning has been recently proposed as a promising tool for fast screening and determination of worst-case heating scenarios of orthopedic implants in MRI enviromnent [16, 17]. Here we report results of a proof-of-concept simulation study to assess the applicability of machine learning to predict RF heating of elongated implants, such as leads in active electronic devices, during MRI at 1.5 T. We tested the hypothesis that a feedforward neural network could be trained to predict the local SAR at tips of implanted leads when only the knowledge of lead’s trajectory within the MRI RF coil and the features of RF coil are at hand. We created clinically relevant lead trajectories analogous to what is observed in patients with cardiac pacemakers/defibrillators to support neural network training. A simple six-layer feedforward neural network with hyperparameters tuned by Ray Tune was shown to be effective in this heat-predicting task as it resulted in a high *R*^2^ score of 0.87 and the RMSE of 14.5 W/kg on the testing set.

Classification tasks of neural networks on MRI safety topics are the future focus. Besides, only typical 58 cm leads were experimented. For more general-purpose, the performance of neural networks with leads in different lengths and number of sample points need to be discussed further.

## Figures and Tables

**TABLE I.** Mesh statistics for a representative simulation

Parts	Num of Tets	Min edge length (mm)	Max edge length (mm)	RMS edge length (mm)
Human body	276366	0.30	26.68	12.62
Cubic region	40625	0.19	2.46	1.57
Insulation	369495	0.11	2.00	0.58
Wire	208258	0.03	1.23	0.42
Coil	38706	9.84	501.79	64.35

## Clinical Relevance

Machine learning can potentially allow real-time assessment of MRI RF safety in patients with conductive leads when only the knowledge of lead’s trajectory (image-based) and MRI RF coil features (vendor-specific) are in hand.
